# Supplementary material for: Virtual epilepsy patient cohort: Generation and evaluation
Source: PLoS Comput Biol. 2025 Apr 11;21(4):e1012911. doi: 10.1371/journal.pcbi.1012911 (PMC12043236; doi:10.1371/journal.pcbi.1012911)
Supplement: S1 Fig — The structure contains the simulated SEEG spontaneous seizures, stimulated seizures and interictal spikes in the main folder of each patient (named sub-001, sub-002, etc.). The parameters used to obtain the simulated time series are detailed in the derivatives folder for each of the EZ hypothesis (either VEP hypothesis or clinical hypothesis). (PDF) [file pcbi.1012911.s001.pdf]

VirtualEpilepticCohort

```
|
|--- sub-001                                                # subject id
|    |--- ses-01                                           # each session corresponds to a simulation type
|    |    |--- ieeg                                        # simulated SEEG in BrainVision format
|    |         |                                           # each run corresponds to one seizure
|    |         |--- sub-001_ses-01_task-simulatedseizure_acq-VEPhypothesis_run-01_ieeg.eeg
|    |         |--- sub-001_ses-01_task-simulatedseizure_acq-VEPhypothesis_run-01_ieeg.vhdr
|    |         |--- sub-001_ses-01_task-simulatedseizure_acq-VEPhypothesis_run-01_ieeg.vmrk
|    |         |--- sub-001_ses-01_task-simulatedseizure_channels.tsv
|    |
|    |--- ses-02
|    |    |--- ...
|    |
|    |--- sub-001_coordsystem.json                          # description on coordinate system
|    |--- sub-001_electrodes.tsv                            # SEEG electrode names and coordinates
|
|--- sub-002
|    |--- ...
|
|--- derivatives                                           # BIDS derivatives format
|    |--- tvb                                              # data simulated using TVB
|         |--- sub-001
|         |    |--- ses-01
|         |    |    |--- VEPhypothesis                     # simulation with VEP hypothesis as EZ
|         |    |    |    |--- parameters                   # model and simulator parameters in TVB
|         |    |    |    |    |--- sub-001_epileptor_parameters_run-01.tsv
|         |    |    |    |    |---  sub-001_simulator_parameters_run-01.tsv
|         |    |    |    |--- img                          #images of the simulated timeseries
|         |    |    |    |    |---  sub-001_simulated_sensor_timeseries_AC_run-01.png
|         |    |    |    |    |---  sub-001_simulated_sensor_timeseries_run-01.png
|         |    |    |    |    |---  sub-001_simulated_source_timeseries_run-01.png
|         |    |    |    |                                  # simulated timeseries on the source level
|         |    |    |    |---  sub-001_simulated_source_timeseries_run-01.tsv
|         |    |    |
|         |    |    |--- clinicalhypothesis                # simulation with clinical hypothesis as EZ
|         |    |         |--- parameters
|         |    |         |--- img
|         |    |         |--- ...
|         |    |
|         |    |--- ses-02
|         |    |    |--- ...
|         |    |
|         |    |--- struct
|         |         |
|         |         |--- sub-001_connectome.zip              # structural connectivity
|         |         |--- sub-001_gain.tsv                    # gain matrix
|         |         |--- img                                 # images of the SC and gain matrix
|         |              |--- sub-001_connectome.png
|         |              |--- sub-001_gain.png
|         |              |--- sub-001_sources_sensors.png
|         |
|         | --- sub-02
|              | --- ...
|
|--- vep_atlas.tsv                                          # mapping node labels to brain region names
|--- participants.tsv                                       # participats id list
|--- dataset_description.json                               # basic dataset metadata (namet, etc.)
|--- README                                                 # description of dataset content and usage
```
