# Supplementary figures and images for: Virtual epilepsy patient cohort: Generation and evaluation
Source: PLoS Comput Biol. 2025 Apr 11;21(4):e1012911. doi: 10.1371/journal.pcbi.1012911 (PMC12043236; doi:10.1371/journal.pcbi.1012911)

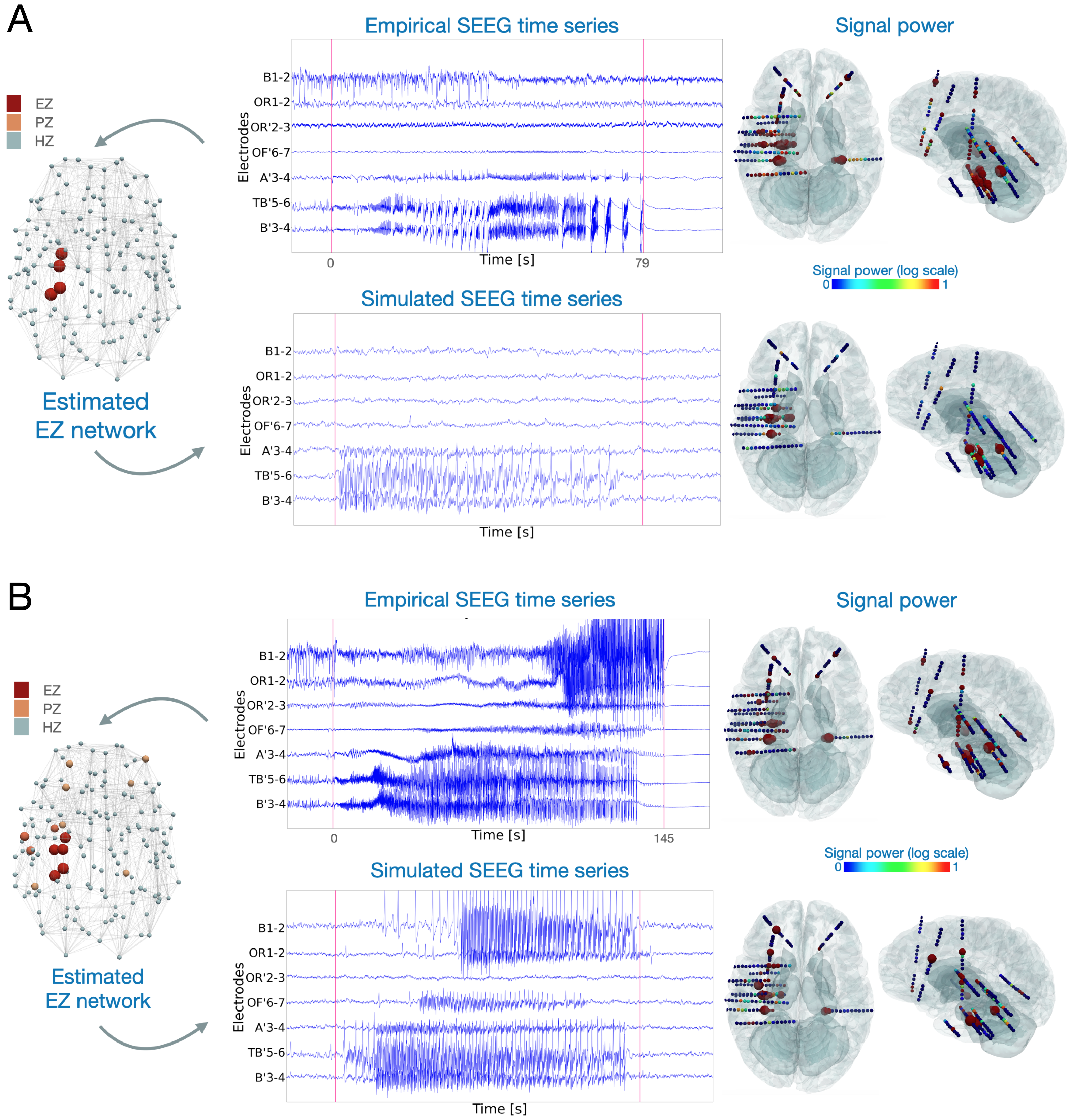

Supplement: S2 Fig — (A) Focal seizure occurring in left temporal lobe. (B) Propagated seizure with onset in left temporal lobe and propagation to contra lateral hemisphere. For both cases, simulated and empirical SEEG seizures are shown. Red vertical lines indicate seizure onset and seizure offset. Signal power for all SEEG channels is shown in 3D in axial and sagittal plane. (PNG) [file pcbi.1012911.s002.png]

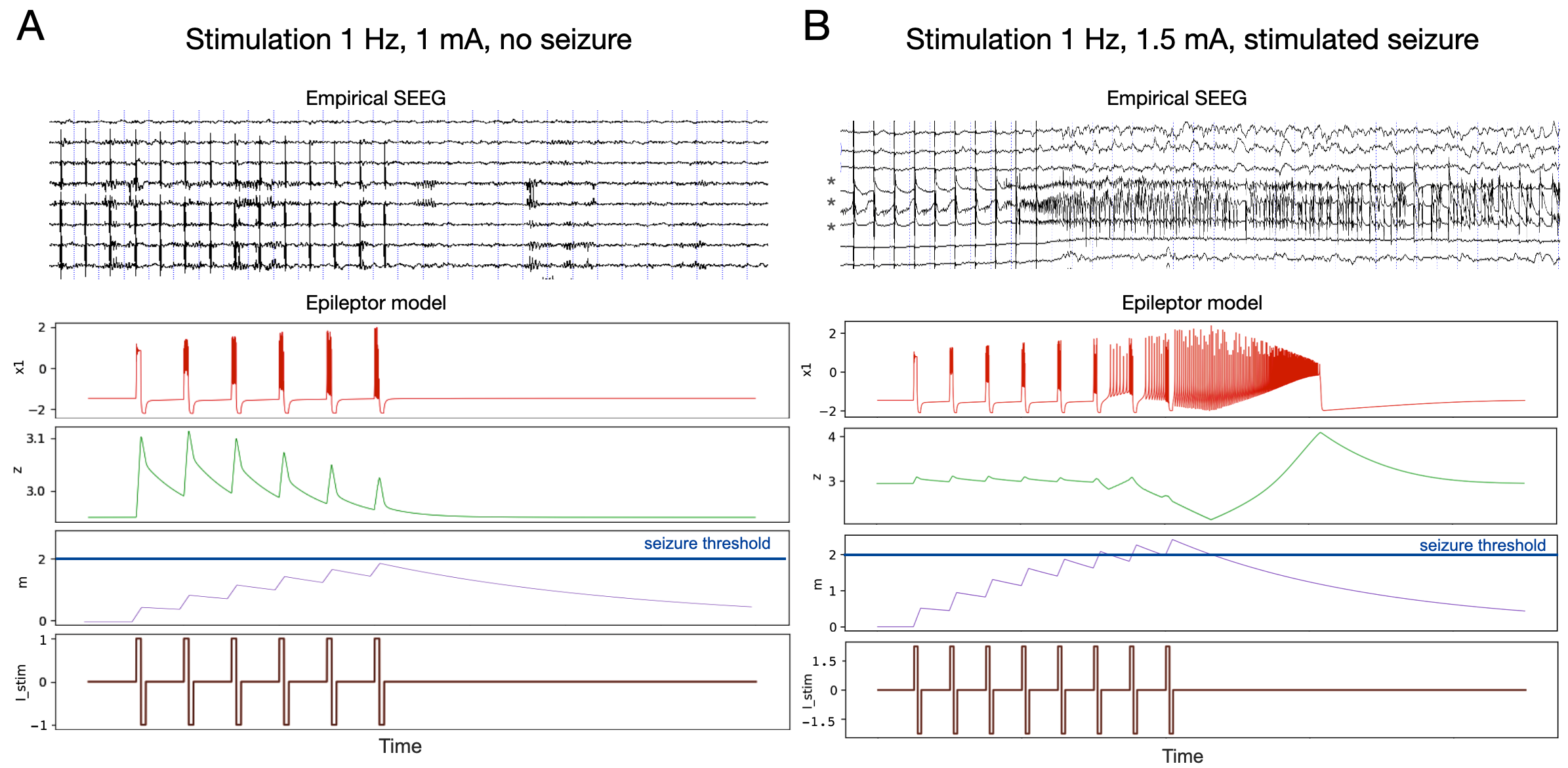

Supplement: S3 Fig — Above, empirical SEEG recording with stimulation artefact present. Below, simulated time series using the Epileptor-stimulation model. Variables of the model x1, z, m and Istim are shown. (A) Example with 1 Hz, 1 mA stimulation and no seizure being induced. (B) Example with 1 Hz, 1.5 mA stimulation and a seizure is induced. Asterisks indicate the time series containing seizure activity. (PNG) [file pcbi.1012911.s003.png]

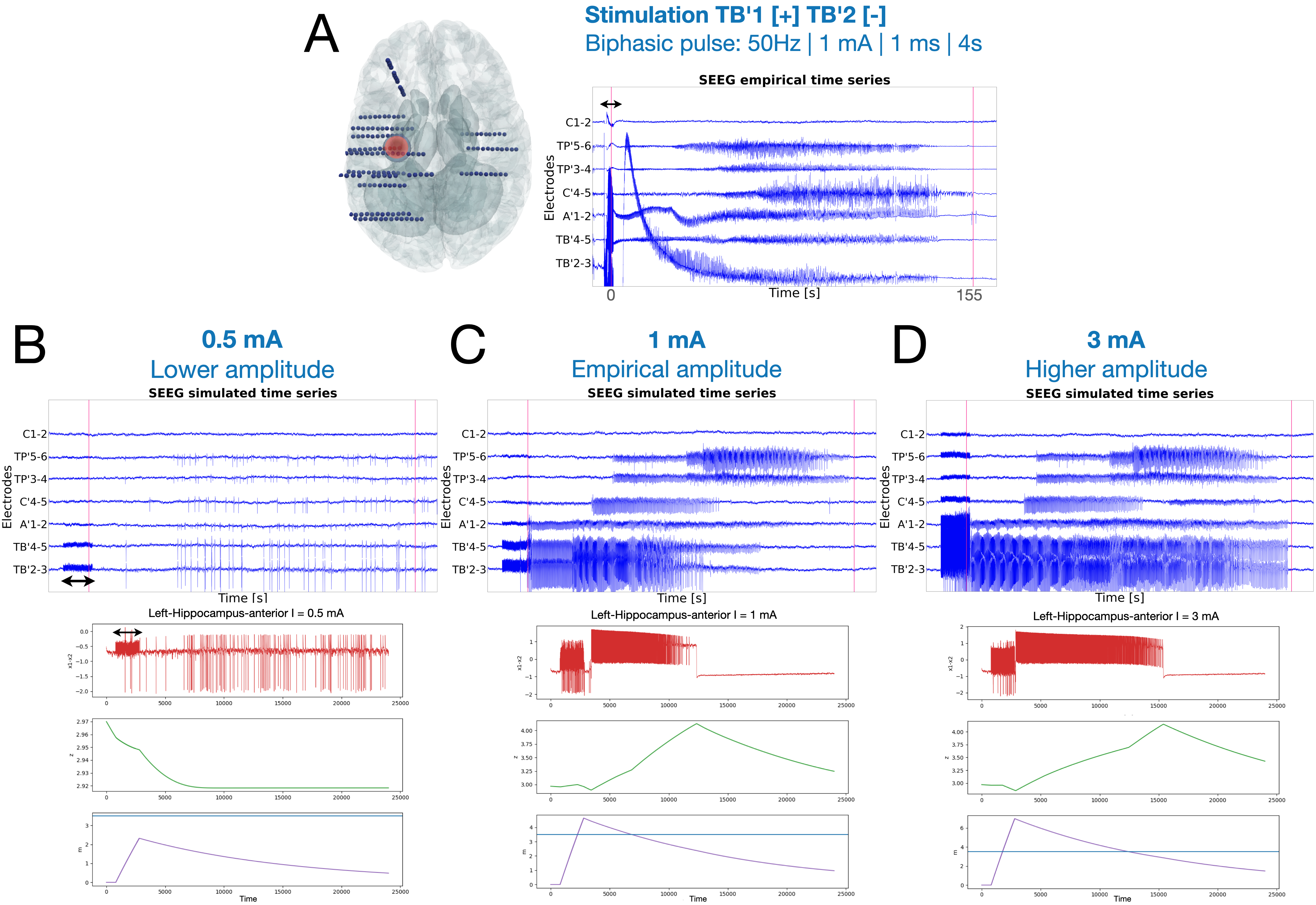

Supplement: S4 Fig — Double arrow indicates stimulation period. Seven channels are plotted in bipolar montage (out of 116 total bipolar channels). Vertical red lines indicate seizure onset and seizure offset. For all plots (B), (C) and (D), upper plots show simulated time series at the SEEG level with stimulation applied at different amplitudes. Lower plots show variables x1−x2 in red, z in green and m in purple evolving for the same simulation for the region left hippocampus anterior. (A) Empirical SEEG recording plot of a stimulation-induced seizure. Stimulation was applied at amplitude 1 mA using channels TB’1 [+] and TB’2 [-], at frequency 50 Hz, pulse width 1 ms and duration 4 s. Reconstructed SEEG electrodes are shown on the left and stimulation location is plotted in red. (B) Upper plot, synthetic SEEG time series of simulated brain activity with stimulation applied at 0.5 mA amplitude. All other stimulation parameters are identical to the empirical parameters. Here, a seizure is not induced after the stimulation is applied. Lower plot, the same simulated activity for the left hippocampus anterior, showing the variable m did not cross the seizure threshold, defined at 3.5 and corresponding variables staying in the normal state. (C) Synthetic SEEG time series of the stimulation-induced seizure at 1 mA amplitude. Here, the same stimulation parameters as the ones applied empirically were used. Following the stimulation, a seizure is induced in the left hippocampus anterior, propagating later on to connected brain structures. Lower plot showing the variable m crossed the seizure threshold and the system is kicked to the seizure state. (D) Synthetic SEEG time series plot of simulated brain activity with stimulation applied at 3 mA amplitude. Following the stimulation, a seizure is induced in the left hippocampus anterior and propagating later on to connected brain structures. Lower plot showing the variable m crossed the seizure threshold and the system is kicked to the seizure state. [file pcbi.1012911.s004.png]

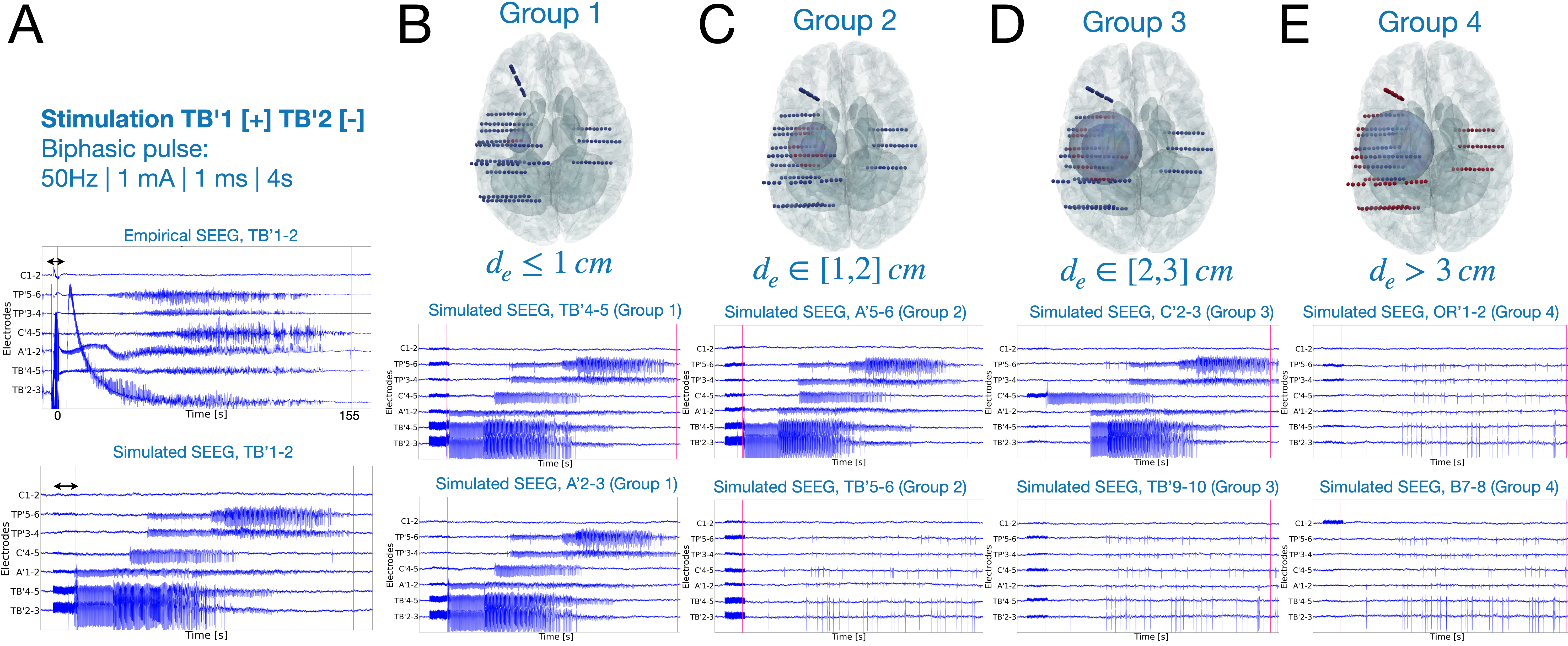

Supplement: S5 Fig — The stimulation location was chosen by randomly selecting a pair of electrodes within a certain radius from the empirical stimulation location. Double arrow indicates stimulation period. Seven channels are plotted in bipolar montage (out of 116 total bipolar channels). Vertical red lines indicate seizure onset and seizure offset. (A) Upper plot, empirical SEEG recording of a stimulation-induced seizure. Stimulation was applied at amplitude 1 mA using channels TB’1 [+] and TB’2 [-], at frequency 50 Hz, pulse width 1 ms and duration 4 s. Lower plot, corresponding simulated time series of a stimulation-induced seizure. (B) Simulated time series of stimulation applied by electrodes located within 1 cm distance from the empirical stimulation location (TB’1-2). (C) Simulated time series of stimulation applied by electrodes located between 1 and 2 cm distance from the empirical stimulation location. (D) Simulated time series of stimulation applied by electrodes located between 2 and 3 cm distance from the empirical stimulation location. (E) Simulated time series of stimulation applied by electrodes located more than 3 cm away from the empirical stimulation location. As the stimulus is applied increasingly further away from the empirical stimulation location, the seizure dynamics progressively changes from the empirical post-stimulation response. (PNG) [file pcbi.1012911.s005.png]

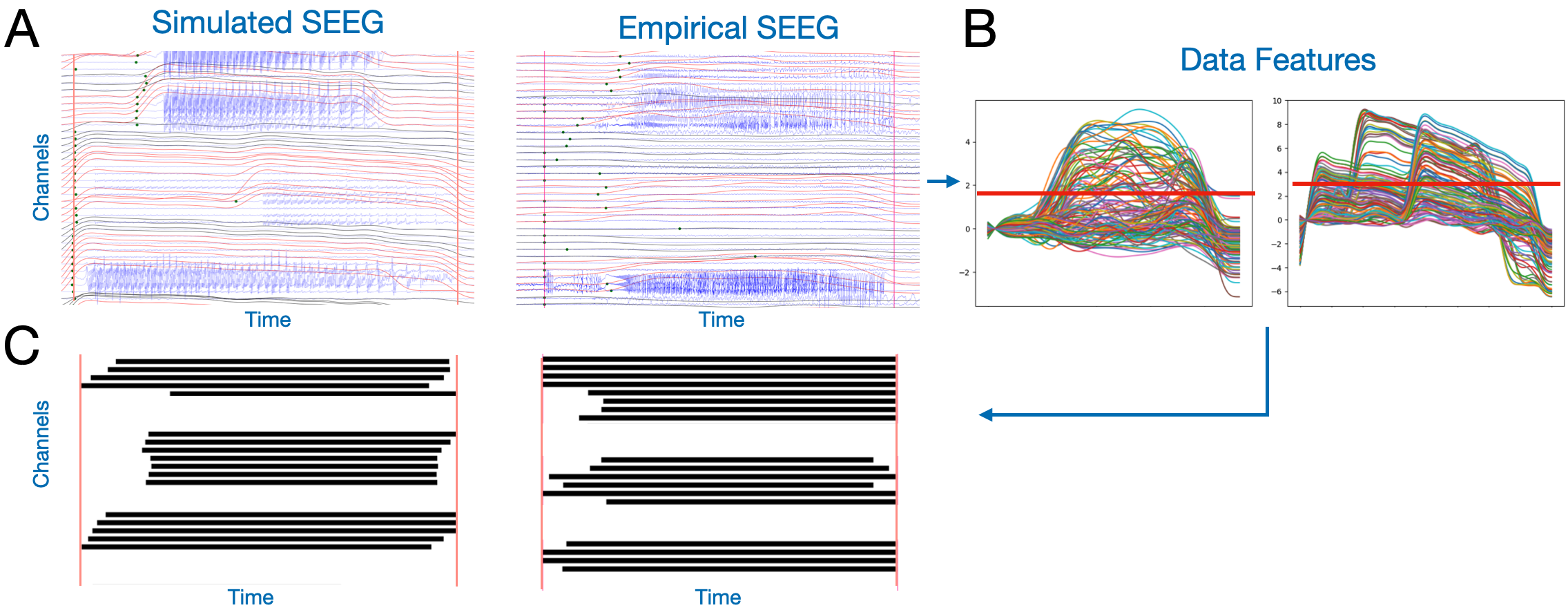

Supplement: S6 Fig — (A) Timeseries plot of a few SEEG channels (right: simulated SEEG time series, left: empirical SEEG time series). Overlayed in red and black are the envelope data features for each SEEG channel, indicating seizure and non seizure channels respectively. Green points indicate estimated seizure onset times. (B) Envelope data features overlayed for all SEEG channels (left: simulated, right:empirical). Horizontal red lines indicate chosen threshold to categorize each channel as either seizure (above threshold) or non-seizure channel (below threshold). (C) Binary plot of the same SEEG channels, where black indicates seizure activity and white indicates no seizure activity. (PNG) [file pcbi.1012911.s006.png]

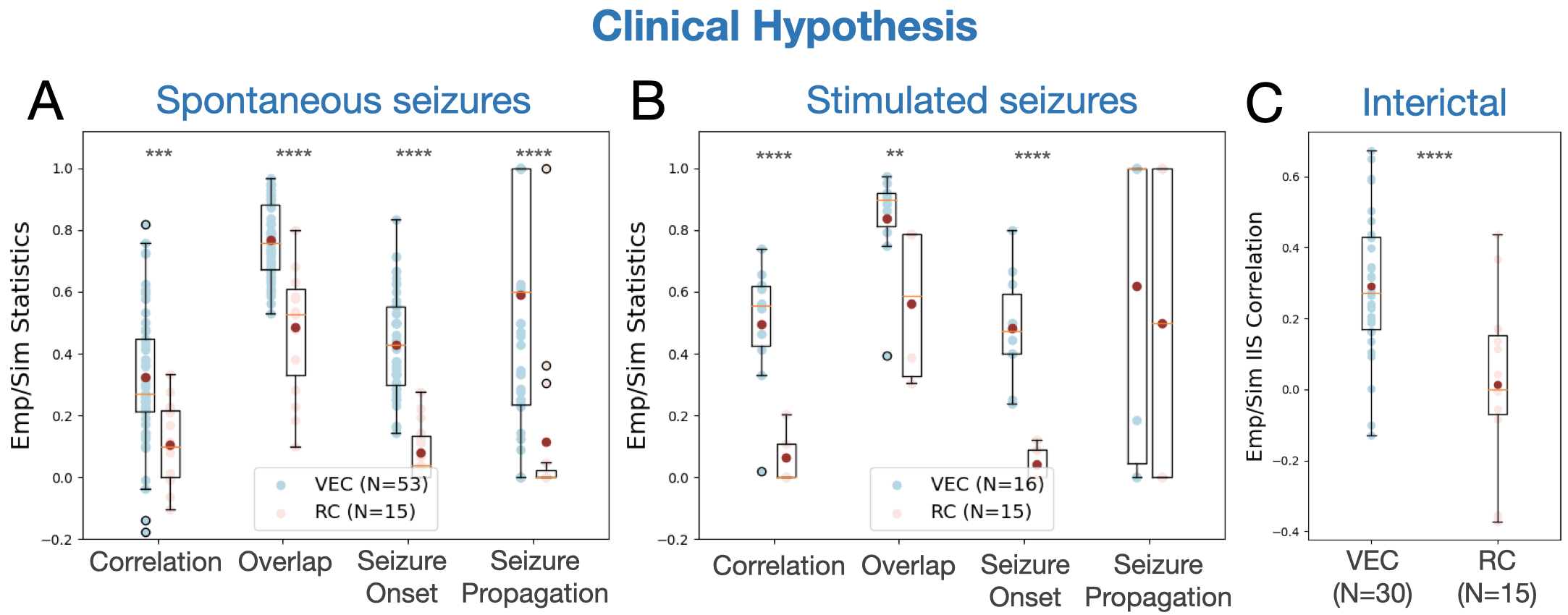

Supplement: S7 Fig — Comparison of simulated SEEG signals with empirical recordings for the virtual epileptic cohort (VEC, in blue) and the randomized cohort (RC, in red). The clinical hypothesis was used to inform the excitability parameters in the model. (A) Boxplot of four main metrics comparing spontaneous seizures against synthetic seizures. Red dots indicate mean values. (B) Boxplot of four main metrics comparing synthetic against empirical stimulation-induced seizures. (C) Boxplot of interictalspike (IIS) count correlation metric. ****p–value < 0.0001, ***p–value < 0.001, **p–value < 0.01; permutation test. (PNG) [file pcbi.1012911.s007.png]

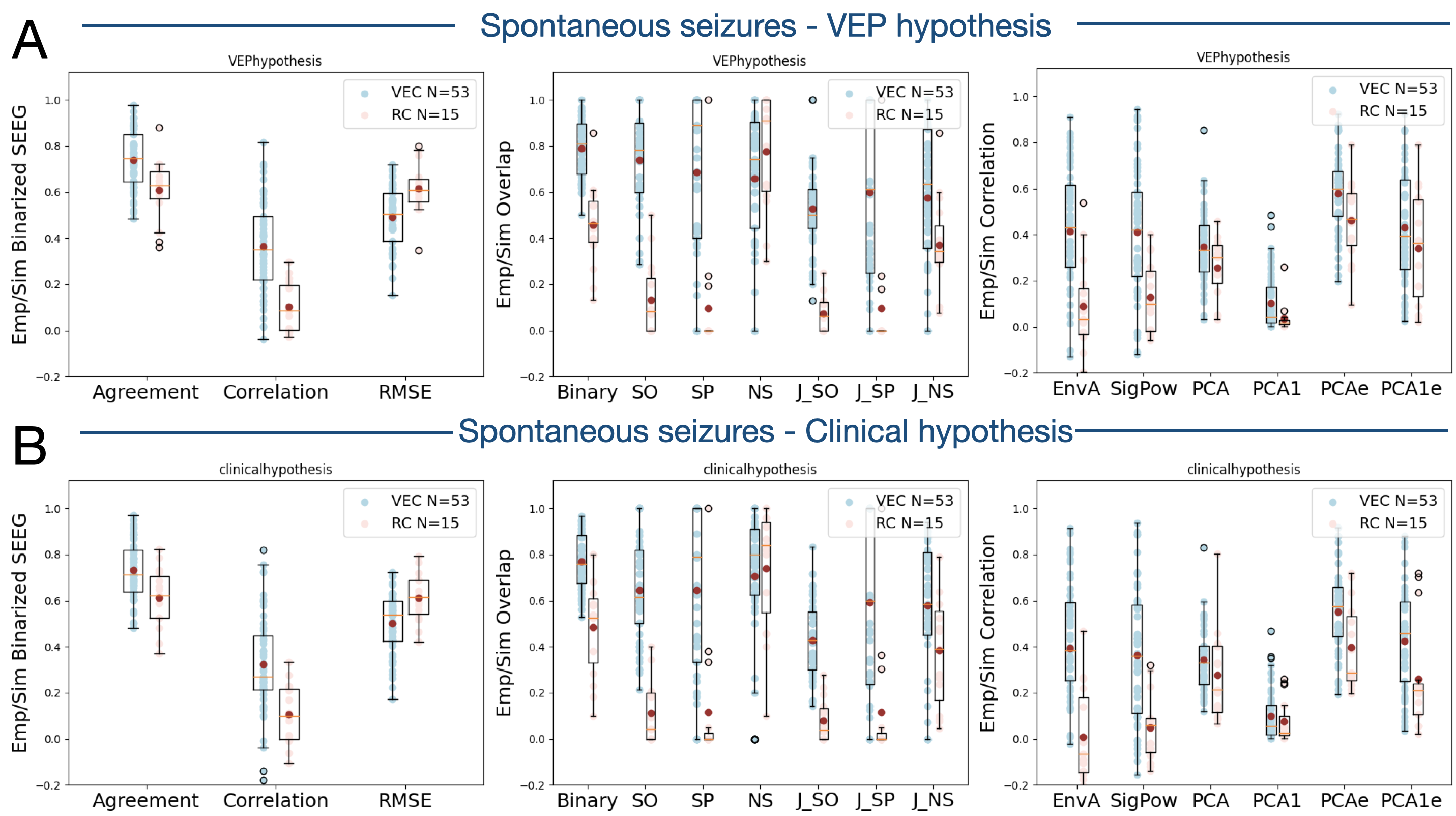

Supplement: S8 Fig — To compare the simulated SEEG time series against the empirical SEEG, sixteen metrics were tested in total. In blue, mean and standard deviation for the VEC cohort. In red, mean and standard deviation for the randomized cohort. (PNG) [file pcbi.1012911.s008.png]

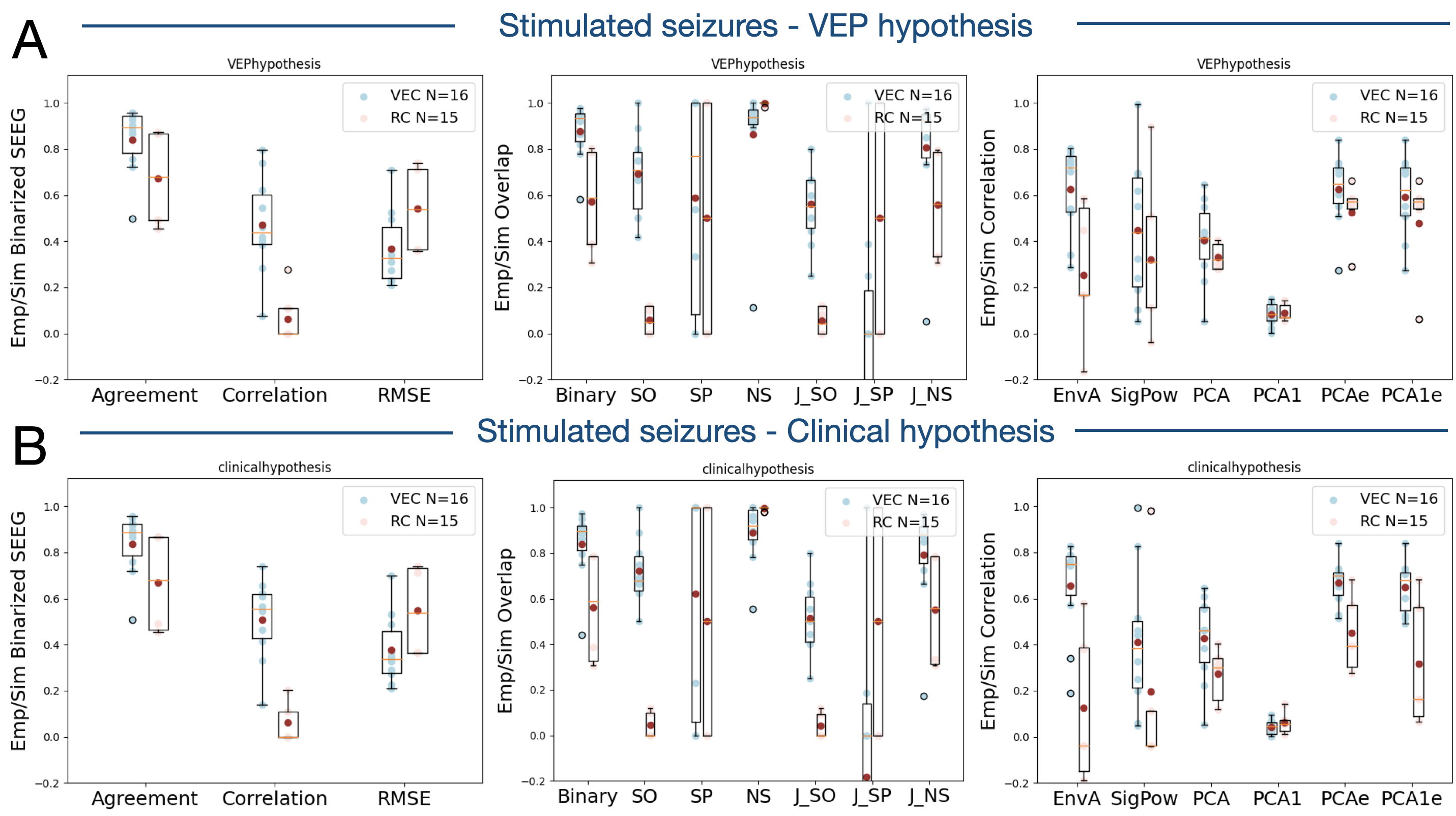

Supplement: S9 Fig — To compare the simulated SEEG time series against the empirical SEEG, sixteen metrics were tested in total. In blue, mean and standard deviation for the VEC cohort. In red, mean and standard deviation for the randomized cohort. (PNG) [file pcbi.1012911.s009.png]

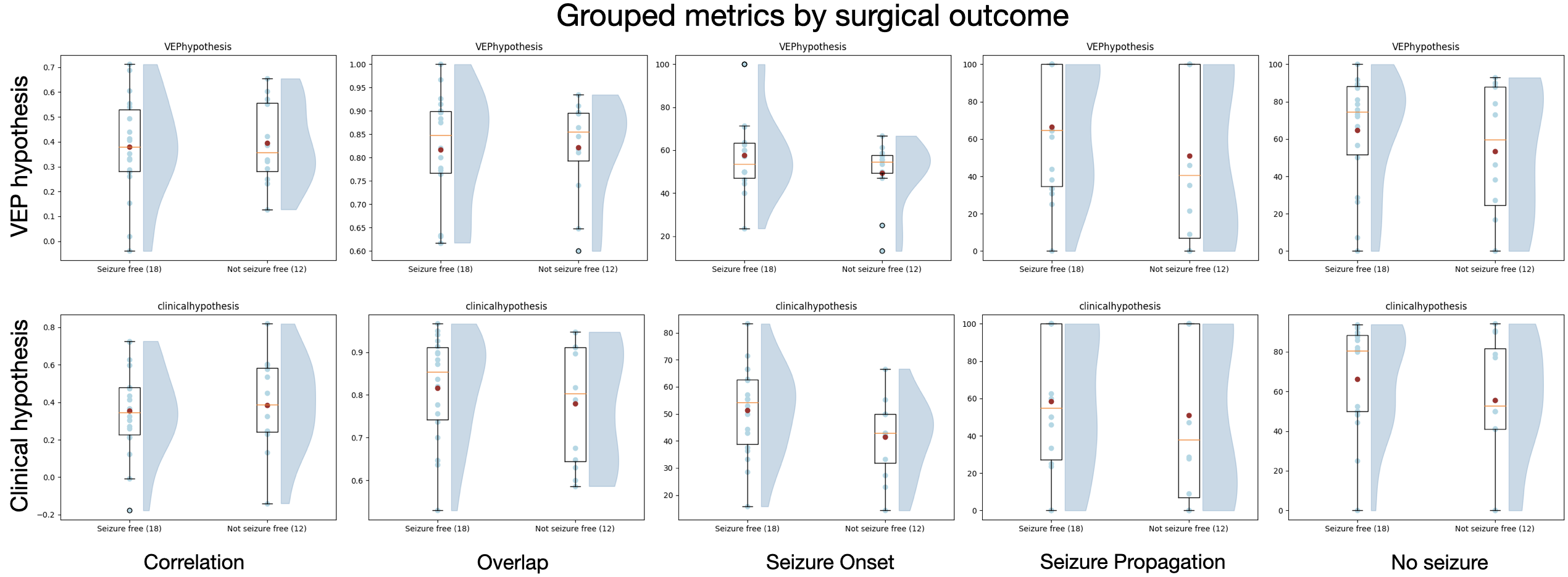

Supplement: S10 Fig — Following their Engel score, patients were grouped in either the seizure-free group (Engel score I) or the not-seizure-free group (Engel scores II, III and IV). First row shows metrics from synthetic data using the VEP hypothesis. The second row uses the clinical hypothesis. In each plot, metrics from seizure-free patients are plotted on the left side, wherease metrics from not-seizure-free patients are plotted on the right side. A boxplot is overlaid over all individual data points and a violin plot from the same data points is shown on the right next to it. (PNG) [file pcbi.1012911.s010.png]
