# Supplementary material for: Virtual epilepsy patient cohort: Generation and evaluation
Source: PLoS Comput Biol. 2025 Apr 11;21(4):e1012911. doi: 10.1371/journal.pcbi.1012911 (PMC12043236; doi:10.1371/journal.pcbi.1012911)
Supplement: S1 File — The file contains a description of the complete set of metrics used to compare simulated and empirical SEEG seizure time series. (PDF) [file pcbi.1012911.s011.pdf]

## Supplementary Material

### Complete set of metrics used to compare simulated and empirical seizures

To compare the simulated SEEG time series against the empirical SEEG, we tested sixteen metrics in total. The metrics are explained in the following subsections, following the same order as in the related figures S8 and S9. In the main article, we selected the following metrics: Binary, Jaccard Seizure Onset, Jaccard Seizure Propagation and Correlation.

#### Overlap metrics

The overlap metric compares, for any given category, the ratio of common SEEG channels between the empirical and simulated sets, divided by the total number SEEG channels in the empirical set. For any given category, it varies between 0 and 1, where 0 indicates no common channels, and 1 indicates that all channels of the empirical set are present in the simulated set.

For both empirical and synthetic SEEG data, we labelled all SEEG channels (in bipolar montage) as either seizure channel ($E_S$, $S_S$ for empirical and simulated case respectively) or no seizure channel ($E_{NS}$, $S_{NS}$ for the empirical and simulated case resp.). A channel is labelled as seizure channel if the envelope data feature crosses the defined threshold, otherwise it is labelled as no seizure channel. The overlap metric used to compare the synthetic and empirical seizure channels is the following:

$$Binary = \frac{|E_S \cap S_S|}{|E_S|} \tag{1}$$

which computes the ratio between the number of common seizure channels between empirical and simulated SEEG, divided by the total number of empirical seizure channels.

In addition, for both empirical and synthetic SEEG data, we labelled all seizure channels as either seizure onset channel ($E_{SO}$, $S_{SO}$ for empirical and simulated case resp.) or seizure propagation channel ($E_{SP}$, $S_{SP}$ for empirical and simulated case resp.). A seizure channel is labelled as seizure onset channel, if the estimated seizure start time in that channel belongs to the first few seconds of the whole seizure (one-fifth of the total seizure window), otherwise the channel is labelled as seizure propagation channel . The overlap metric used to compare synthetic and empirical data for each category (seizure onset, seizure propagation and no seizure) is as follows:

$$SO = \frac{|E_{SO} \cap S_{SO}|}{|E_{SO}|} \quad SP = \frac{|E_{SP} \cap S_{SP}|}{|E_{SP}|} \quad NS = \frac{|E_{NS} \cap S_{NS}|}{|E_{NS}|} \tag{2}$$

The Jaccard similarity coefficient compares the similarity between to sets, by also taking into account the number of non-common items between the two sets (by computing the union between the two sets). It varies between 0 and 1, where 0 indicates the sets have no elements in common, and 1 indicates the sets are identical. The Jaccard similarity coefficient applied to each category (seizure onset, seizure propagation and no seizure) is as follows:

$$SO_{Jaccard} = \frac{|E_{SO} \cap S_{SO}|}{|E_{SO} \cup E_{SO}|} \quad SP_{Jaccard} = \frac{|E_{SP} \cap S_{SP}|}{|E_{SP} \cup E_{SP}|} \quad NS_{Jaccard} = \frac{|E_{NS} \cap S_{NS}|}{|E_{NS} \cup E_{NS}|} \tag{3}$$

where $E_{SO}$, $E_{SP}$ and $E_{NS}$ are the empirical seizure onset, seizure propagation and no seizure channels, respectively. $S_{SO}$, $S_{SP}$ and $S_{NS}$ are the synthetic seizure onset, seizure propagation and no seizure channels, respectively.

#### Correlation metrics

The sample Pearson correlation coefficient is used for all the metrics of this section, where the paired data $(s_1, e_1), ..., (s_n, e_n)$ consisting of $n$ SEEG channels, is used to compute $r$. The coefficient varies between $-1$ and $1$.

$$r = \frac{\sum_{i=1}^{n}(s_i - \bar{s})(e_i - \bar{e})}{\sqrt{\sum_{i=1}^{n}(s_i - \bar{s})^2}\sqrt{\sum_{i=1}^{n}(e_i - \bar{e})^2}}$$

For both simulated and empirical cases, the envelope peak amplitude is computed for each SEEG channel. Then, the sample Pearson correlation between the two lists of amplitude values is computed, $EnvA$.

For both simulated and empirical cases, the signal variance is computed for each SEEG channel. The sample Pearson correlation between the two lists of variance values is computed.

$$V = \frac{1}{T}\sum_{t=1}^{T}(x_t - \bar{x})^2 \tag{4}$$

For both simulated and empirical cases, PCA is performed on the data. First the SEEG timeseries are standardized for each channel, by substracting the mean and dividing by the standard deviation. Then, PCA is performed on the time series using the same number of components as the number of SEEG channels. Then, the Pearson correlation is computed between the main principal components (i.e. the ones that explain 90% of the variance) of the empirical and simulated data, thus obtaining a correlation matrix. From this matrix, the maximum correlation value is extracted as a comparative value, $PCA$. As a second approach, the Pearson correlation is computed between the two first components of the empirical and simulated data, $PCA1$.

For both simulated and empirical cases, PCA is performed on the envelope data features, using the exact same approach as above, but replacing the SEEG time series by the envelope time series. The metrics computed are $PCA_e$ and $PCA1_e$.

### Comparative metrics for 2D binarized SEEG

We performed four main comparative metrics on the binarized images ($E_{bin}$ for the empirical case, $S_{bin}$ for the simulated case) obtained from the SEEG time series.

First, we performed an overlap metric by measuring the number of identical binary (simulated, empirical) pairs divided by the total number of pixels for one image.

$$agreement = \frac{|E_{bin} \cap S_{bin}|}{|E_{bin}|} \tag{5}$$

Second, we performed a Pearson $correlation$ on the 2D images, by comparing each binary (simulated, empirical) pair of identical row and column.

Third, we computed the mean squared error and the root mean squared error between the two binary images, with total number of $N$ values:

$$mse = \frac{1}{N}\sum_{i=1}^{N}(E_{bin}(i) - S_{bin}(i))^2 \quad rmse = \sqrt{mse} \tag{6}$$

## Grouping metrics by surgical outcome

We grouped the patients of the VEC cohort according to the surgical outcome in two groups: seizure-free and not-seizure-free. In figure S10, we plot the metrics performance for these two groups. These data were pooled following the Engel score of each patient, where patients with Engel score I were assigned the seizure-free group and patients with Engel scores II, III and IV were assigned the not-seizure-free group. This analysis was purely performed for exploratory purposes. Our interpretation follows that since our purpose was to build sythetic data that best match the empirical recordings, we seem to have little to no bias in whether the data came from patients that were seizure-free or not.
