# Supplementary material for: Virtual epilepsy patient cohort: Generation and evaluation
Source: PLoS Comput Biol. 2025 Apr 11;21(4):e1012911. doi: 10.1371/journal.pcbi.1012911 (PMC12043236; doi:10.1371/journal.pcbi.1012911)
Supplement: Attachment — Submitted filename: Response_to_reviewers___VEC_paper_PCOMPBIO.pdf [file pcbi.1012911.s012.pdf]

Notes on revision made to PLOS Computational Biology Manuscript no. PCOMPBIOL-D-24-01804

We would like to thank the editor and reviewers for their constructive comments and suggestions that have really helped improve the quality of this manuscript. We have carefully considered all comments and revised the manuscript accordingly to address the reviewers' concerns. Please see below our responses. For the reviewers' convenience, our responses are provided below in blue, and all significant changes in the revised manuscript are highlighted in red.

Response to the reviewers

Reviewer 1

Reviewer Comment 1.1 — General:

The authors present a synthetic dataset of 30 virtual epileptic patients. This is potentially useful to the community studying seizures and resective surgery. The manuscript is well presented, providing a thorough description of the methods used to generate and analyse the data.

The aim of the manuscript is clearly to present the dataset and illustrate capabilities, rather than to present new results. The presentation of interictal spikes and stimulus evoked seizures is novel only within the VEP framework, but again presents a potentially useful resource for investigating the links between seizures and stimulus evoked dynamics. I would encourage the authors to expand on the points below to improve the presentation of the context of the work.

Reply: Thank you very much for your thoughtful and encouraging feedback. Please find below our responses to each of your comments.

1 Major Issues: Methodological

Reviewer Comment 1.2 — The VEP framework is not a universal ground truth for the generation of seizures nor the evaluation of epilepsy surgery. It would be of benefit to explore this more thoroughly in the manuscript. For example, though the assumptions are implicit in the description of the model, it would be beneficial to end users to spell these out upfront, in the main part of the manuscript, interpret them biologically and critique them. For example, in this model, simulated seizures are due to a build up in "z", which is designed to push nodes through bifurcations into seizures (in what sense are they spontaneous? Is recurrent a better description?). This is only one of a few possible models of recurrent or spontaneous seizure generation, none of which have been shown to be universally true (see work by Lopes da Silva, Piotr Suffczynski, Gerold Baier etc..). Similarly, in this model, propagation patterns emerge due to the distribution of the excitability parameter, and the seizure onset zone is equated to the epileptogenic zone. This may or may not be the case, see Lopes et al. 2019 (https://www.frontiersin.org/journals/computational-neuroscience/articles/10.3389/fncom.2019.00025/full) for a computational evaluation of the role of network structure and the interplay with excitability. These are important points to highlight since

the synthetic data will be useful for exploring methods to detect the EZ only for cases in which these assumptions hold, and readers should be appropriately informed.

Reply: Thank you so much for this valuable and helpful comment. We agree with your points raised above and try to clarify them in the manuscript.

(i) Indeed, the VEP framework is a proposed methodology, based on Bayesian inference, which estimates the posterior distributions of epileptogenicity of brain regions of a personalized whole brain model based on observations of empirical SEEG seizure recordings. This framework is currently used in a clinical trial and has been evaluated for a subset of patients in Wang et al. Sci Trans Med 2023. The aim of this approach is to complement the clinician's perspective when diagnosing the epileptogenic zone (EZ) using computational models, with the end goal of improving surgical outcome for people suffering from focal drug-resistant epilepsy.

(ii) Secondly, the VEP framework uses the Epileptor model when building personalized whole brain models to simulate spatio-temporal seizure dynamics. The excitability parameter x0 of the model is inferred based on observed SEEG seizure data, in addition to the global network coupling parameter K. This is our model of choice, however other phenomenological models for seizure dynamics exist in the literature (Saggio et al, Da Silva, Suffczynski etc.). The choice of the Epileptor model is based on (i) the control parameter x0 which is directly linked to regional epileptogenicity, (ii) the simplified 2D Epileptor model offering a low-dimensional model for whole-brain seizure dynamics allowing for an efficient inference of the EZ from empirical seizure data, (iii) the permittivity coupling between Epileptors which predicts whole-brain seizure emergence based on regional excitability and patient-specific structural connectivity.

(iii) Finally, the estimated EZ from the VEP framework is used to parametrize the x0 parameter for each regional Epileptor on the virtual brain model of a patient. In addition, the structural connectivity is used to determine the connection weights between each Epileptor, allowing for simulations at the whole-brain level to be performed, where seizure emergence depends on both x0 and network connectivity. Therefore, our approach does not contradict Lopes et al. 2019. In our paper, we have measured the seizure onset and seizure propagation in simulated SEEG data and compared it against empirical SEEG data.

In summary, in order to clarify the assumptions of our approach, we have added the following sentences in the manuscript, which we have combined with the previous second paragraph in the discussion section:

When using the virtual epileptic cohort data, there are several important points to take into account. First, the VEP framework for EZ hypothesis estimation is a proposed methodology undergoing evaluation in a clinical trial, with some preliminary results (Wang et al., 2023). It constructs a whole brain model for a given patient using individual brain imaging and Bayesian inference methods to specify the parameters from seizure data. Second, simulated spontaneous (i.e. unprovoked recurrent) seizures are driven by the slow dynamics of the permittivity variable z in the Epileptor model, which guides the system periodically from normal to seizure-like states. This variable mimics time-dependent changes during seizures as extracellular $[K^+]$, oxygen and ATP consumption (Jirsa et al., 2014). Studies have shown that the transition to seizure is a slow process, involving gradual loss of network resilience (Chang et al., 2018; Rich et al., 2022), increased sensitivity to stimulation (Jiruska et al., 2010) and ion dynamics changes, including extracellular $[K^+]$ (Fröhlich et al., 2008), intracellular chloride (Lillis et al., 2012) and extracellular calcium (Wenzel et al., 2019), although ionic processes are highly intertwined (Raimondo et al., 2015). However, no universal mechanism is known for seizure generation and several other dynamical models for seizure onset have been proposed (Da Silva et al., 2003; Suffczynski et al., 2004; Wang et

al., 2014; Goodfellow et al., 2011; Wendling et al., 2005; Baier et al., 2012; Breakspear et al., 2006; Saggio and Jirsa, 2023). Thirdly, we hypothesized that repetitive stimulation causes slow accumulation of ion imbalances which can trigger seizures. To model stimulated (i.e. provoked) seizures, we made m time-varying in the Epileptor model, which influences the stability of the seizure state (El Houssaini et al., 2020). During stimulation, m slowly increases until crossing a seizure threshold, pushing the system into the seizure state (see supplementary Figure S3). The seizure threshold was tuned based on the EZ hypothesis and empirical stimulation parameters. Lower stimulation amplitudes failed to cross the threshold, whereas empirical and higher amplitudes succeeded (see supplementary Figure S4). In future studies, this approach can be refined for each patient when more empirical stimulation data are integrated for model inversion. Finally, for both types of simulated seizures, generation of seizure dynamics on a virtual brain depends on the EZ hypothesis and the structural brain connectivity (SC). The EZ hypothesis informs the excitability parameter x_0 for each regional Epileptor, while the SC determines the connection weights between them, such that regional brain activity depends on both its excitability and the activity of connected regions (Lopes et al., 2019; Proix et al., 2017). This allows synthetic seizures to emerge as a whole-brain phenomenon influenced by local activity and connectivity.

Reviewer Comment 1.3 — Figure 1 shows the first example of stimulated seizures. It would be important to clarify in what sense these are 'seizures', rather than, for example, afterdischarges. For example in Gollwitzer et al. 2018 (https://pubmed.ncbi.nlm.nih.gov/30269940/) the term afterdischarges is used, with seizures emerging from these in only 3.5% of cases. Ley et al. 2022 (https://www.sciencedirect.com/science/article/pii/S1388245722001699) provides some useful background, and considers stimulation evoked activity to be 'seizures' if they are the same as a patient's seizures electrographically and have the same semiology. Furthermore they note that afterdischarges are not necessarily a reliable marker of the seizure onset zone (or epileptogenic zone), which implies that the mechanisms (underlying network and excitability patterns) producing these and seizures may not be the same, i.e. may not be reproducible with the same underlying structural connectivity or distributed excitability in the model. Again, this should be clarified for the reader who is interested in using the presented synthetic data to understand stimulation evoked activity.

Reply:

Thank you for sharing your insights and the two references.

The stimulated seizures coming from the retrospective dataset have been marked by expert epileptologists, and afterdischarges (ADs) have not been taken into account here. We use a similar definition to ADs and seizures as in Ley et al. 2022 and Gollwitzer et al. 2018, but also Trebuchon et al. 2016. Citing Ley et al. 2022: *"ADs were defined as rhythmic transient epileptic activity caused by DCS, which does not produce clinical symptoms and shows no evolution but lasts for more than 2 seconds"*.

However, we do recognize that we have not separated True Positive (TP) seizures and False Positive (FP) seizures as Ley et al. 2022 defines them. They consider stimulated seizures to be TP when they are identical to spontaneous seizures, otherwise they are considered to be FP. This is in accordance with the French school definitions, which call TP seizures "habitual seizures", which are indeed considered to be targeting structures closer to the EZ.

Nevertheless, for the patient ID 08 shown in Response Figure 1, there are two types of spontaneous seizures (also shown in supplementary Figure S2), and we have only shown one of the two. The second type of seizure occurs over a sub-network of the first type, which corresponds to the stimulated seizure, see Figure below (Response Figure 1). Here below, we show two spontaneous seizures of different types of that patient. The spontaneous seizure which did not have complex propagation patterns (Response

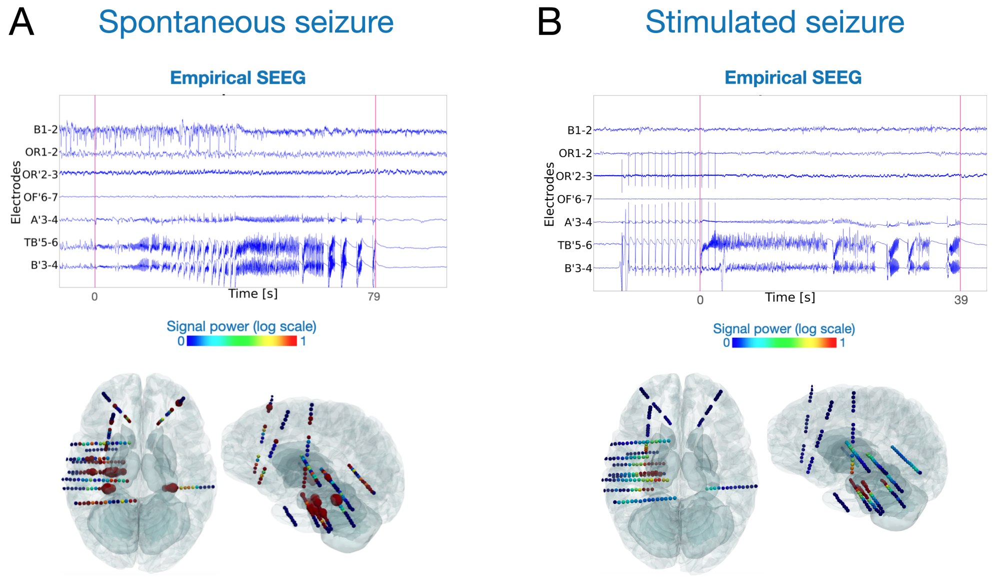

Response Figure (1): Comparison of spontaneous seizure and stimulated seizure of patient ID 08. SEEG recordings (upper panels) and signal power across SEEG electrodes during the seizure time window (lower panels). A) Spontaneous seizure. B) Stimulated seizure.

Figure 1A) matches from an electrographic point of view the patient's triggered seizure (Response Figure 1B).

We have added the following sentences in the manuscript to clarify whether we used ADs in our dataset and true positive / false positive seizures: "Stimulated seizures in the empirical dataset were marked by expert epileptologists. Additional stimulations which did not trigger seizures or triggered afterdischarges (ADs) were not simulated. ADs are generally not considered to be a reliable predictor of the seizure onset zone and may have different underlying mechanisms from seizures (Ley et al. 2022, Gollwitzer et al. 2018). In addition, stimulated seizures resembling a patient's spontaneous seizures, both electrographically and in semiology, are strong predictors of postsurgical outcomes (Ley et al., 2022). In our dataset, semiology was not considered, and all stimulated seizures were simulated without distinction. "

Reviewer Comment 1.4 — The data consists of seizures, evoked 'seizures' and spikes. This is motivated in the introduction since these types of activity can be used to constrain the EZ. The relationship between the EZ, spikes and evoked activity is not straightforward though and background dynamics are also being shown to contain information useful for delineating the EZ [e.g. Taylor et al. 2022 https://academic.oup.com/brain/article/145/3/939/6514463].

Reply: Thank you for your comment. We agree that we do not cover all the complexity of the epileptic brain in the resting state and that there are many more features to take into account. We also agree that the relationship between these three modalities is still unclear; here we have provided a simple modeling approach where the underlying EZ network (inferred via the VEP pipeline or provided by clinicians) is used to provide all three modalities.

We add a sentence in our discussion section in our manuscript to clarify this: The relationship between seizures, spikes, and stimulated activity is not straightforward. Our VEP cohort can be a tool

for researchers to verify their hypotheses across these three modalities. However, we do not address the full complexity of the epileptic brain in the resting state and background dynamics, which have been shown to contain useful information for delineating the EZ (Taylor et al., 2022).

Reviewer Comment 1.5 —

"Here we present for the first time synthetic seizures triggered by SEEG stimulation" in introduction and "Here, we present for the first time..." in discussion are a bit of a stretch I think. Many previous models have presented stimulus evoked seizures (see e.g. Baier et al. 2017 https://www.frontiersin.org/journals/computational-neuroscience/articles/10.3389/fncom.2017.00025/full and wider literature).

Reply: Thank you for your comment, we fully agree. We have revised these sentences in the manuscript.

In introduction section:

To account for synthetic seizures triggered by SEEG stimulation, we extended the Epileptor model.

In discussion section:

Here, we presented synthetic stimulated seizures by extending the Epileptor model, which captures spatio-temporal seizure dynamics and interictal spikes (Jirsa et al., 2014).

Reviewer Comment 1.6 — The section 2.1 could be improved by adding further (brief) details on the comparisons that were made, for example a brief account of what it means to reproduce the clinically defined EZ with a precision of 0.6.

Reply: Following your comment, we have added further details to section 2.1.

" The VEP hypothesis was evaluated retrospectively by comparing the inferred EZ with the clinical hypothesis and surgical resection. For all patients, the VEP accurately reproduced the clinically defined EZ network with a mean precision of 0.6, showing significant overlap. Despite some discrepancies, the physical distance between their epileptogenic regions was small (mean, 5.67 mm) (Wang et al., 2023). Additionally, the VEP was compared to the resected brain regions of 25 patients who underwent surgery. Here, the false discovery rate (FDR) was employed, to measure the proportion of falsely identified EZ regions (i.e. which were not resected) over all identified regions. The VEP hypothesis showed lower FDR in seizure-free patients (mean, 0.028) compared to those who were not seizure-free (mean, 0.407). This suggests that the VEP could improve predictive power, especially in non-seizure-free cases.

Reviewer Comment 1.7 — In section 2.4.1 it would be useful to clarify to what extent the stimulation parameters are the same as those used clinically. The model is not biophysical so the real electrical disturbance cannot be recreated.

Reply: Following your remark, we have added further details to section 2.4.1.

We used the EZ hypothesis derived from spontaneous seizures and modeled the electrical stimulus based on the clinical stimulation parameters. Specifically, the spatial distribution of the electric field was computed based on the stimulation location and charge, while the stimulus waveform was modeled according to the stimulation frequency, pulse width, and total duration. Finally, the strength of the electric field was input into the Epileptor-stimulation model, scaled proportionally to the values of its phenomenological variables. The scaling parameter is determined by comparing empirical signals with simulated ones (see Equation 4).

Reviewer Comment 1.8 — The end of the paragraph in discussion starting "We simulated.." contains a section on neural field models that only refers to the authors' previous work. I would suggest to widen this to include work from other members of the community.

Reply: Following your comment, we have included works from other members of the scientific community.

Conversely, neural field models describe neural activity dynamics on finer spatial scales ($\sim 1\,mm^2$ of the cortical surface) and consider short-range cortical connections in addition to long-range white matter connectivity (Amari, 1977; Wilson and Cowan, 1973; Coombes, 2005; Hutt & and Atay, 2005; Jirsa, 2009; Bressloff, 2011).

Reviewer Comment 1.9 —

Finally, although it is potentially useful to present synthetic data, the community would benefit hugely from sharing of the actual recordings. There are precedents for this - e.g. databases with surgery records and EEG from Mayo and MGH (at https://www.ieeg.org/) also see Wong et al. 2023 (https://pmc.ncbi.nlm.nih.gov/articles/PMC10235576/). It would be interesting to hear why the authors have chosen not to share the original EEG, if indeed that is the case.

Reply:

We acknowledge the reviewer's point and appreciate their attention to the importance of data sharing to advance research in the community. We fully recognize the value of publicly accessible SEEG datasets. However, sharing raw SEEG data from France is challenging due to strict legal and ethical regulations, including compliance with the General Data Protection Regulation (GDPR) in the EU and oversight by the Comission Nationale de l'Informatique et des Libertés (CNIL) in France. These regulations ensure the protection of patient privacy and confidentiality. As such, the legal framework in France requires a case-by-case review and approval process for sharing patient data.

To address this, we have instead provided synthetic data in the form of a virtual cohort. This dataset mimics key characteristics of the original data while safeguarding patient privacy and adhering to regulatory requirements. We believe this approach strikes a balance between advancing research and respecting the legal and ethical constrains imposed on patient data sharing. We hope this clarifies our decision and welcome further suggestions.

Reviewer 2

Reviewer Comment 2.1 — The Ms describes a dataset of synthetic seizures generated in 30 virtual epilepsy patients, using patient-specific anatomical and functional connectivity data and the epileptor model to simulate seizure dynamics. The dataset is publicly available, and the manuscript argues that this synthetic cohort can serve as a valuable resource for evaluating data analysis methodologies. The primary advantage of synthetic seizures is the availability of ground truth regarding the epileptogenic zone (EZ), which is often unavailable in empirical data. Overall, I have no major concerns with the manuscript, but I provide several comments below that should be addressed before it is ready for acceptance. Addressing these points will mainly enhance the clarity and presentation of the manuscript.

Reply: Thank you for your feedback. We will carefully revise the manuscript to incorporate your suggestions and ensure it meets the standards for acceptance.

Reviewer Comment 2.2 — Figure 1 Workflow Consistency: In Figure 1, the simulated seizures are presented in the upper panel, while empirical seizures appear below. In contrast, Figures 2, 4, and 6 reverse this order, showing empirical signals above simulated ones. Is there a specific reason for this reversal? Does it reflect a difference between the workflow for generating the virtual cohort and the synthetic signals depicted in Figures 2, 4, and 6?

Reply: No, there is no difference between the workflow for generating the virtual cohort in Figure 1 and the synthetic signals in Figures 2, 4 and 6. We have rectified this by consistently showing the empirical data first and the simulated data second. For this, we modified the order in Figure 1 of the manuscript.

Reviewer Comment 2.3 — 2. EZ Hypothesis Clarification: Figure 1's caption states that the EZ hypothesis, derived from ictal SEEG data, informs the excitability parameter for each brain region. The study uses two EZ hypotheses: one based on the Virtual Epileptic Patient (VEP) hypothesis from spontaneous seizures, and another clinically defined by experts. Please clarify which hypothesis is used in the Figure 1 workflow and whether this aligns with the depicted sequence (simulated seizures first, empirical seizures second).

Reply: Thank you for your comment. We have clarified this in the figure's caption:

The EZ hypothesis informs the excitability parameter for each brain region and can be derived from ictal SEEG data. Simulated data shown here were generated using the EZ hypothesis from the Virtual Epileptic Patient (VEP) pipeline.

Reviewer Comment 2.4 — 3. Seizure Propagation Metrics: The manuscript mentions that the performance for the virtual epileptic cohort is significantly better than the randomized cohort ($p < 0.001$), as shown in Figure 5A. However, please also note that Seizure Propagation metrics showed no differences between the two cohorts.

Reply:

Thank you for your comment. We raised this point in the Discussion section:

However, the seizure propagation metric (SP) did not perform as well for stimulated seizures compared to spontaneous seizures, highlighting a limitation of our study.

In addition, we have added the following sentence in the Results section 2.4.2 when referring to Figure 5A:

The results demonstrate a significantly better performance for the virtual epileptic cohort compared to the randomized cohort, as shown in Figure 5A. However, here the seizure propagation metrics (SP) showed no significant differences between the two cohorts.

Reviewer Comment 2.5 — 4. Boxplot Presentation Issues: 4.1 Some boxplots depicting cohort statistics appear odd and need improvement. For example, in Figure 3D, the VEC boxplot for "Seizure Propagation" shows most points below the median except for two. Similarly, in Figure 5A for the RC boxplots, the middle box contains only two points, though it should include 50% of the data (approximately 7 or 8 points). This may be due to overlapping points. If it so, I suggest to overlay box plots with swarm plots.

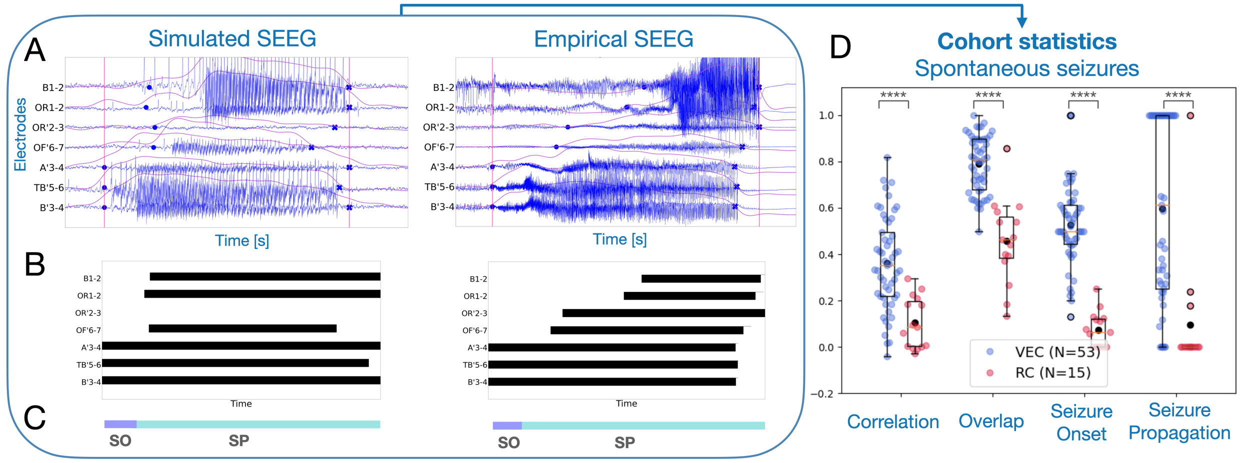

Response Figure (2): Updated figure 3 of the main manuscript.

4.2 The RC cohort points have a very light color shade, making them hard to discern. Consider using a stronger shade for better visibility.

4.3 The dark red point in each boxplot, representing the mean value, is described as "dark red" but appears as just a darker shade of the RC cohort points. This is misleading, as it also represents the mean for the VEC. Using a different, unrelated color (e.g., black or violet) would help avoid confusion.

Reply: We have edited the Figure 3D of the main manuscript following your comments, as depicted below in Response Figure 2.

We also edited Figure 5A similarly in the main manuscript, as depicted below in Response Figure 3. For consistency, we also updated Figure 6C in the main manuscript as depicted in Response Figure 4.

Reviewer Comment 2.6 — 5. Redundant Paragraph: The paragraph in lines 88–98 includes information that overlaps with earlier descriptions in lines 79–87, such as the use of synthetic data for validating analysis methods and the role of the virtual brain twin. Please revise to reduce redundancy.

Reply: We have revised our manuscripts according to your suggestions. Now we have kept a single paragraph, which we updated to avoid this redundancy. We also placed a sentence of the second paragraph in a previous paragraph of the introduction. All changes are highlighted in red in the main manuscript.

We place the combined paragraph here as it is also present in the introduction section:

As a result, we generated a virtual cohort of 30 drug-resistant epilepsy patients. To account for synthetic seizures triggered by SEEG stimulation, we extended the Epileptor model. Each virtual brain twin contains synthetic spontaneous seizures, stimulated seizures and interictal activity at the whole-brain level and at the SEEG level. The novelty of our work is the generation of a comprehensive virtual patient cohort, enabling researchers to evaluate their methods using synthetic data that mimic empirical data. Each patient's estimated EZ serves as model parameters, providing its known ground-truth from the modeling setting and detailed parameters. We introduced metrics to systematically compare this virtual cohort against the corresponding empirical cohort. Additionally, we interrogated the influence

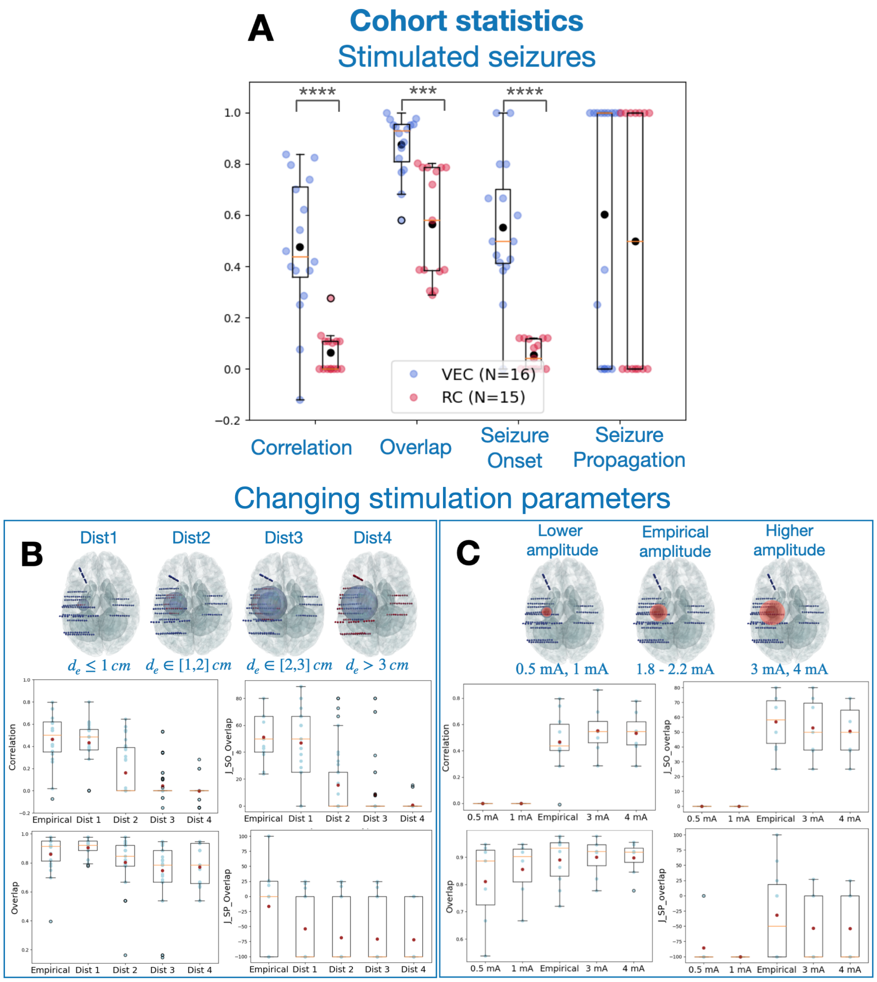

Response Figure (3): Updated figure 5 of the main manuscript.

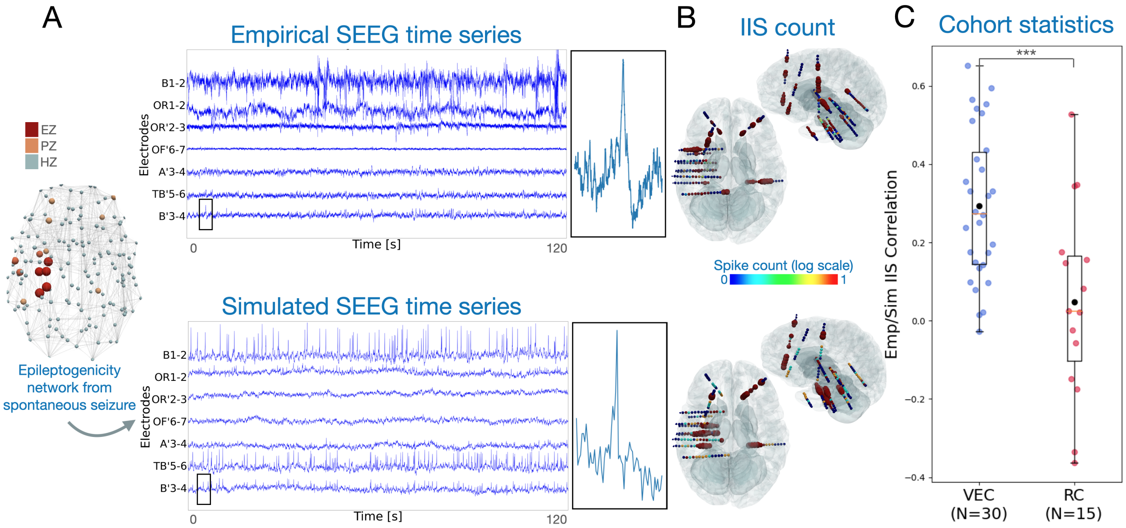

Response Figure (4): Updated figure 6 of the main manuscript.

of stimulation location and simulation amplitude on seizure networks. We shared this dataset publicly in iEEG-BIDS format (Holdgraf et al., 2019) to help the scientific community when systematically evaluating and refining their EZ estimation methods.

Reviewer Comment 2.7 — 6. Acronym Clarification: The acronym VEC is introduced in line 152 but only explained in the Figure 3 legend. Please define it upon first use.

Reply: Thank you for your comment. We have rectified this upon the first use of the acronym:

Examples of spontaneous seizures from two patients of the virtual epileptic cohort (VEC) are shown in Response Figure 2.

Reviewer Comment 2.8 — 7. Parameter Explanation: The meaning of the parameters k and q in Equation (5) is not explained. Additionally, it appears that k in Equation (5) differs from k in Equation (4). Please clarify.

Reply: Thank you for your comment and apologies to not have clarified this. In Equation (5), $k = \frac{1}{4\pi\epsilon}$. In order to use k only once in Equation (4), we have replaced the expression of k in Equation (5) by the formula it equates to as follows:

$$|\vec{E}(\vec{r})| = |\frac{q}{4\pi\epsilon}(\frac{\vec{r} - \vec{r_{q+}}}{|\vec{r} - \vec{r_{q+}}|^3} - \frac{\vec{r} - \vec{r_{q-}}}{|\vec{r} - \vec{r_{q-}}|^3})| \quad (1)$$

We edited the section 4.7 in the manuscript as follows:

The field magnitude at a brain location $\vec{r}$ and permittivity ϵ is computed as:

$$|\vec{E}(\vec{r})| = |\frac{q}{4\pi\epsilon}(\frac{\vec{r} - \vec{r_{q+}}}{|\vec{r} - \vec{r_{q+}}|^3} - \frac{\vec{r} - \vec{r_{q-}}}{|\vec{r} - \vec{r_{q-}}|^3})| \quad (2)$$

where ϵ is the permittivity of the brain medium. We set $\epsilon = 1$, as the electric field magnitude input to the phenomenological model is scaled based on the range (minimum and maximum) of the model variables.
